# The Role of Widefield and Ultra Widefield Optical Coherence Tomography in the Diagnosis and Management of Vitreoretinal Diseases

**DOI:** 10.3390/diagnostics12092247

**Published:** 2022-09-17

**Authors:** Matteo Ripa, Lorenzo Motta, Teresa Florit, Jean-Yves Sahyoun, Veronika Matello, Barbara Parolini

**Affiliations:** 1 Ophthalmology Unit, Fondazione Policlinico Universitario A. Gemelli IRCCS, 00168 Rome, Italy; 2 Ophthalmology Unit, Catholic University “Sacro Cuore”, 00168 Rome, Italy; 3 Department of Ophthalmology, William Harvey Hospital, East Kent Hospitals University NHS Foundation Trust, Ashford TN24 0LZ, UK; 4 Department of Ophthalmology, Eyecare Clinic, 25124 Brescia, Italy; 5 Department of Ophthalmology, Université de Montréal, Montreal, QC H3T 1J4, Canada; 6 Department of Ophthalmology, Centre Hospitalier de l’Université de Montréal (CHUM), Montreal, QC H2X 3E4, Canada

**Keywords:** choroidal lesions, high myopia, peripheral lesions, peripheral retinoschisis, swept-source optical coherence tomography, retinal detachment, ultrawide-field optical coherence tomography, vitreoretinal diseases, wide-field optical coherence tomography, wide-field optical coherence tomography angiography

## Abstract

Background: This study reports on the advantages of wide-field (WF)- and ultra-widefield (UWF)- optical coherence tomography (OCT) and optical coherence tomography angiography (OCTA) in managing different vitreoretinal diseases in a real-life setting using the new WF—Swept Source (SS)—OCT Xephilio S1 (Canon, Tokyo, Japan). Methods: We conducted an observational retrospective case series study involving 1472 eyes that underwent retinal scans with Canon Xephilio^®^ OCT-S1 between 1 March 2021 and 1 December 2021 at Eyecare Clinic (Brescia, Italy). All patients underwent routine ophthalmologic examinations along with WF and UWF color fundus retinography with Clarus 500™ (Carl Zeiss Meditec, Inc., Dublin, CA, USA) and Xephilio^®^ OCT-S1. WF SS-OCT, UWF-OCT, WF-OCTA, and UWF-OCTA were taken by using Xephilio^®^ OCT-S1. Results: We analyzed 122 peripheral retinal lesions, 144 retinal detachment, 329 high myopic eyes, 37 pediatric cases, 60 vascular retinopathies, 15 choroidal lesions, and 90 eyes as follow-up post vitreoretinal surgery. The OCT-S1 was the only reliable and diagnostic exam for peripheral lesions, pediatric and high myopic cases, and significantly influenced the management in 10% of cases and the postoperative follow-up. Conclusions: WF and UWF OCT and OCTA imaging may help in the management of several vitreoretinal diseases, becoming an indispensable tool for the high-quality management of patients.

## 1. Introduction

Optical coherence tomography (OCT) and OCT angiography (OCTA) are widely used in evaluating patients with macular and vitreoretinal interface diseases. [1,2].

Huang et al. published the first retinal OCT images in 1991 [3], and OCT has advanced considerably over the last three decades. Notably, OCT instruments have been widely used in ophthalmology since the commercial introduction of the spectral domain (SD)-OCT in 2006 due to the SD-OCT’s higher speed and sensitivity over the time-domain (TD)-OCT. Indeed, its faster image capture and higher definition enabled volumetric imaging with superior image quality [4,5,6]. Subsequently, the third-generation OCT, swept-source (SS)-OCT, incorporated many significant technological advancements. SS-OCT enabled faster scanning speed (100 kHz) and greater tissue penetration, allowing imaging and quantitative evaluation of the choroid for the first time [4,5,6,7,8], as well as better penetration through cataracts and other media opacities by combining swept-source technology with a longer wavelength light source. In addition, SS-OCT enabled vitreous imaging while maintaining clear visibility of the choroid in the same scan [9,10,11]. Wide-field (WF) SS-OCT has recently revolutionized the imaging of vitreoretinal disorders, obtaining images with enlarged fields-of-view (FOV) of 80° compared to the 30° of the standard OCT systems. Furthermore, scans of the peripheral retina are acquired by moving the eye in different positions, resulting in an ultra-wide FOV.

OCTA is a non-invasive imaging technique based on OCT technology that allows visualization of the eye’s blood vessels and red blood cell movement detection, thus demonstrating vascular bed distribution [12]. Despite the advantages, the limited FOV and eye motion artifacts still represent the main challenge [2]. The recently introduced WF-OCTA overcomes these limitations by providing an enlarged FOV and thus yielding peripheral vascularization information [13,14].

The new WF SS-OCT Xephilio S1 (Canon, Tokyo, Japan) with a 1060 nm wavelength can capture up to 23 × 20 mm WF-OCTA images with a single photograph in a limited time (100,000 A-scans/second) and leads to an enhanced view from the vitreous to the sclera offering a better penetration through media opacities, thus yielding outstanding tomographic images.

Our study aimed to report the advantages of WF- and UWF-OCT and OCTA in managing different vitreoretinal diseases in a real-life setting using the new WF SS-OCT Xephilio S1. Therefore, we assessed the “effectiveness” of WF- and UWF-OCT and OCTA in evaluating vitreoretinal diseases whose diagnosis could not be achieved with any other clinical or instrumental examination. Furthermore, we evaluated their “effectiveness” in terms of faster but reliable diagnosis in challenging cases such as pediatric, high myopic, non-cooperative patients or patients’ eyes that had recently undergone vitreoretinal surgery.

## 2. Materials and Methods

### 2.1. Patients

In this observational retrospective case-series study, we collected data from 1472 eyes that underwent retinal scans (WF-OCT, UWF-OCT, WF-OCTA, and UWF-OCTA) with Canon Xephilio ^®^ OCT-S1 and with Clarus 500™, (Carl Zeiss Meditec, Inc., Dublin, CA, USA) between 1 March 2021 and 1 December 2021 at Eyecare Clinic (Brescia, Italy). Data from patients who underwent retinal scans were retrieved and collected. This study followed the tenets of the Declaration of Helsinki for research involving human subjects. Informed consent was obtained from all subjects to use the data for the study. All images of patients with vitreous abnormalities (floaters, opacities, and cellularity), peripheral retinal lesions, retinal detachment, and choroidal and optic nerve lesions were included. In addition, images of challenging cases (miosis, severe media opacity, pediatric cases, postoperative follow-up) were also grounds for inclusion. However, poor OCT or OCTA images due to severe media opacity or unstable fixation were excluded.

### 2.2. Examinations and Image Acquisition Protocol

All patients underwent routine ophthalmologic examinations, including the measurement of corrected distance visual acuity (CDVA) in the logarithm of the minimum angle of resolution (logMAR), the measurement of intraocular pressure (IOP) with a non-contact tonometer, a slit lamp bio-microscopy, and a dilated fundus evaluation using 90 diopters (D) lens. Afterwards, a single trained technician took all retinal images comprised of color fundus retinography, standard macular OCT, and widefield OCT.

WF and UWF color fundus retinography were obtained with Clarus 500™, (Carl Zeiss Meditec, Inc., Dublin, CA, USA). The Clarus 500™ fundus image was acquired by a Confocal Scanning Laser Ophthalmoscope (cSLO) with partial confocal optics. Using an internal fixation light, a single image and two fundus images were recorded from two different horizontal visual angles. These two images were automatically merged to create a montage image with a wide field of view.

The Xephilio^®^ OCT-S1 (Scanning Laser Ophthalmoscope, SLO: 855 nm) was obtained for each patient using the three-dimensional (3D) macula scan with a maximum scan width of 10 mm. The confocal image was obtained alongside each OCT examination. Based on OCT scanning, the projection image was obtained from collecting all the A-scans and the reflectivity of the various layers. Specifically, the WF SS-OCT, UWF-OCT, WF-OCTA, and UWF-OCTA were taken using Xephilio^®^ OCT-S1. In more depth, all patients were imaged using single-capture twelve 10 mm cross-sectional radial scan patterns at 15° intervals. The scanned line length was 10 mm in the horizontal and vertical directions, and the scan depth was 2.0 mm. Cross-sectional scans and long radial scans centered on the fovea were obtained. The map scans were performed with 256 single horizontal scans in the region of interest. In addition, the WF-OCTA images were obtained by scanning areas of different sizes that ranged from small areas (3 × 3 mm, 10°) to super large areas (23 × 20 mm, 80°). A UWF-OCTA was obtained by collecting different images and creating a mosaic of 31 × 27 mm (110°). All images were extracted as JPG files and converted to tiff format. Afterwards, two independent investigators (B.P and L.M) collected and analyzed all images. Both the investigators were blinded to the retinal findings of the patients.

### 2.3. Image Grading

Two OCT specialists (B.P. and L.M.) blinded to the subject’s retinal findings examined all images according to a pre-specified protocol. If consensus could not be reached, a third specialist (M.R.) was consulted for the final decision. The protocol evaluated either the presence or the absence of five parameters: motion (image interruptions), decentration (center of scan not aligned with the center of the macula), defocus (reduced retinal reflectivity due to poor autofocus), masking (light blockage due to anterior or posterior ocular abnormalities such as vitreous opacities and pigment clumps, which do not permit the beam to reach deeper layers), and segmentation errors (unclear boundary of the different layers of the retina). Images with at least one artifact were discarded.

Finally, the images were classified according to the main diagnosis. However, some eyes were affected by multiple conditions; therefore, the number of diagnoses exceeded the number of eyes.

### 2.4. Statistical Analysis

The interobserver agreement was calculated using a kappa (κ) statistic [9]. The κ statistics were calculated and assessed as following: <0.20, poor; 0.21–0.40, fair; 0.41–0.60, moderate; 0.61–0.80, substantial; and 0.81–1.00, almost perfect agreement.

## 3. Results

### 3.1. Demographics

Demographics and categories are listed in Table 1 and Table 2. The mean examination times per different types of scans in cooperative patients are reported in Table 3. All patients underwent WF and UWF color fundus retinography, standard macular OCT, and WF-OCT. The UWF-OCT, WF-OCTA, and UWF-OCTA were performed in 21%, 40%, and 3% of the eyes, respectively. The interobserver agreement between the two examiners was not inferior to 0.93 for each comparison.

### 3.2. Vitreous Pathologies

#### 3.2.1. Floaters and Posterior Vitreous Detachment (PVD)

Floaters were detected as hyper-reflective images with non-homogenous patterns on OCT and dark shadows on the WF-SLO image. Only 131 of 644 eyes with floaters reported symptoms. WF-OCT detected a complete PVD in 571 (39%), partial PVD (detached from macula or papilla) in 69 (5%), and no PVD in 560 eyes (38%). The vitreous cavity appeared homogeneous and optically empty in 272 (18%) vitrectomized eyes.

WF-retinography could neither detect floaters nor PVD. Standard macular OCT allowed suspecting a complete PVD in 200 eyes (14%) to diagnose a partial PVD (detached from macula or papilla) in 50 (3%) and suspected no PVD in 230 eyes (16%). The vitreous cavity appeared homogeneous and optically empty in 272 (16%) vitrectomized eyes. 

#### 3.2.2. Anomalous PVD

**Figure 1:**
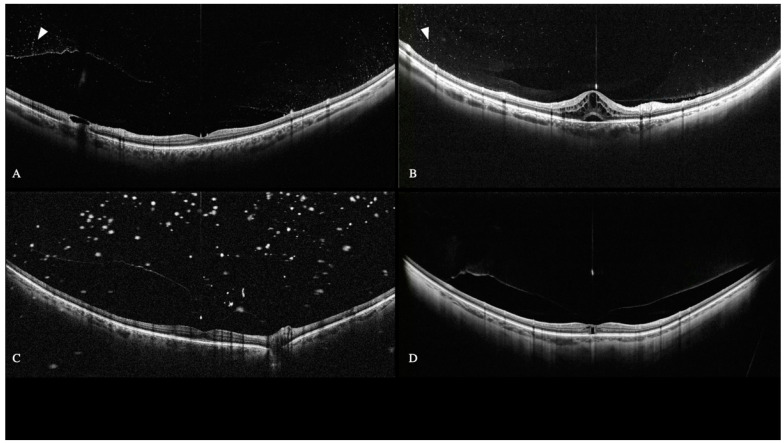
WF SS-OCT shows vitreous cellularity (arrowheads) in toxoplasma retinitis (**A**) and bilateral macular and optic nerve edema secondary to Behçet disease (**B**). Asteroid hyalosis induces bigger and hyper-reflective vitreous opacities (**C**). Vitreomacular Traction with subfoveal outer lamellar macular hole and initial foveal schisis. The vitreous is attached to the central fovea and at the equator (**D**).

OCT-S1 detected the location and extension of vitreoretinal anomalous adhesions and tractions (Figure 1).

#### 3.2.3. Vitreous Opacities and Cellularity

Eleven eyes with various densities of vitreous hemorrhage (VH) underwent WF-OCT scans.

Retinography, standard macular OCT, and slit lamp biomicroscopy could not thoroughly examine the fundus. Blood’s cellularity was perceived as a collection of numerous aggregate dots (Figure 1). The vitreous cellularity in five uveitic eyes was remarkably similar to that originating from blood. Furthermore, the cellularity could be neither detected with retinography nor standard macular OCT.

Six eyes were diagnosed with asteroid hyalosis (AH). WF-OCT detected these anomalies in the vitreous as multiple hyperreflective dots with posterior shadowing (Figure 1C). AH’s cellularity size was larger than that related to inflammation and hemorrhage.

No significant differences were found on OCT between inflammatory vitreous cellularity and VH (Figure 1). Indeed, both inflammatory and hemorrhage cells appeared as small and aggregated dots.

### 3.3. Peripheral Retinal Lesions and Retinal Detachment

#### 3.3.1. Peripheral Lesions

UWF-OCT was acquired in 311 eyes (21%). In this subgroup, 39% had peripheral lesions, and 31% were not detectable with slit lamp biomicroscopy, WF retinography, and standard macular OCT.

#### 3.3.2. Retinal Detachment

WF and UWF-OCT revealed the location and extent of the retinal detachment (RD), the retinal tears location, and preretinal membranes in all eyes with RD.

**Figure 2:**
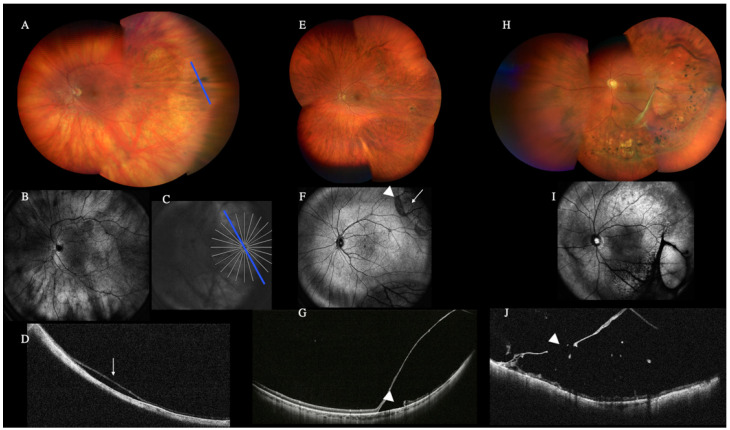
Color fundus retinography (**A**) and scanner laser ophthalmoscopy (SLO) image (**B**,**C**) of a peripheral temporal retinal detachment that could only be diagnosed on the wide-field optical coherence tomography (WF-OCT) (**D**) (arrow). Peripheral retinoschisis is visualized in the color fundus retinography (**E**) and SLO image (**F**). The two different areas seen in the SLO image (**F**) allowed us to differentiate the retinoschisis area (arrow) from the retinal detachment area (arrowhead). With the WF-OCT (**G**), an outer retinal break and a contiguous area of retinal detachment (arrowhead) can be seen. This limited retinal detachment was missed with the fundus retinography. Color fundus retinography (**H**) SLO image (**I**) and WF-OCT (**J**) of a peripheral retinoschisis with vitreous tractions and internal retinal break (arrowhead) that could be better visualized with WF-OCT.

WF-retinography could also demonstrate the presence of RD in all 36 primary and 108 recurrent RD. WF retinography and standard OCT did not always detect the tears and peripheral lesions (Figure 2).

### 3.4. High Myopia

Only WF-OCT could detect the posterior staphyloma’s presence, depth, and perimeter. Specifically, the projection image could better see the edge of the staphyloma as a dark ring.

**Figure 3:**
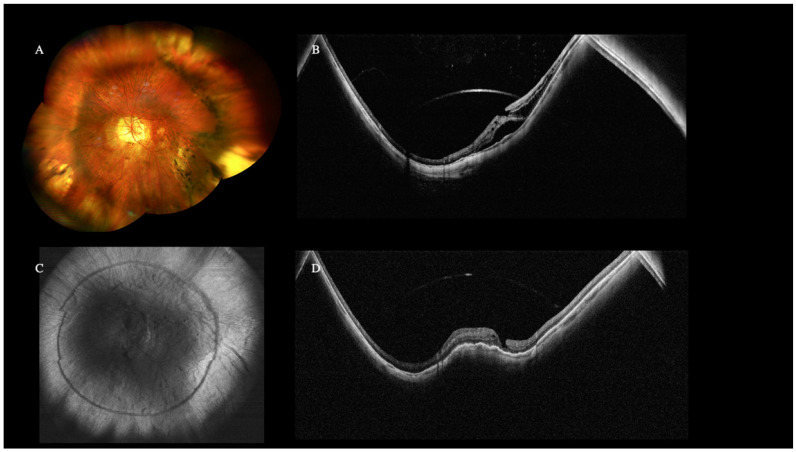
Wide-Field (WF) color fundus retinography of pathologic myopia and posterior staphyloma (**A**). The wide-field optical coherence tomography (WF-OCT) image (**B**) shows the posterior staphyloma and the choroidal thinning in these eyes. Myopic Traction Maculopathy (MTM) stage 3b was diagnosed. The OCT (**C**) projection image shows the staphyloma’s perimeter. The patient underwent a macular buckle that resolved the retinal detachment and the macular schisis (**D**).

When compared with WF-retinography, the edge of the staphyloma was easily visualized with the projection image as a dark ring (Figure 3).

Myopic Traction Maculopathy (MTM) was diagnosed in 28% of high myopic eyes, and the stage was identified in all MTM cases, according to the MTM Staging System [15]. Due to the height of the detachment and the depth of the staphyloma, standard field OCT could not depict 30% of the cases of MTM, particularly in stages 3 and 4 with foveal and macular detachment. At the same time, the WF-OCT could completely illustrate them. Neither WF-retinography nor slit lamp biomicroscopy could detect the MTM’s presence and stage.

### 3.5. Choroidal Lesions

**Figure 4:**
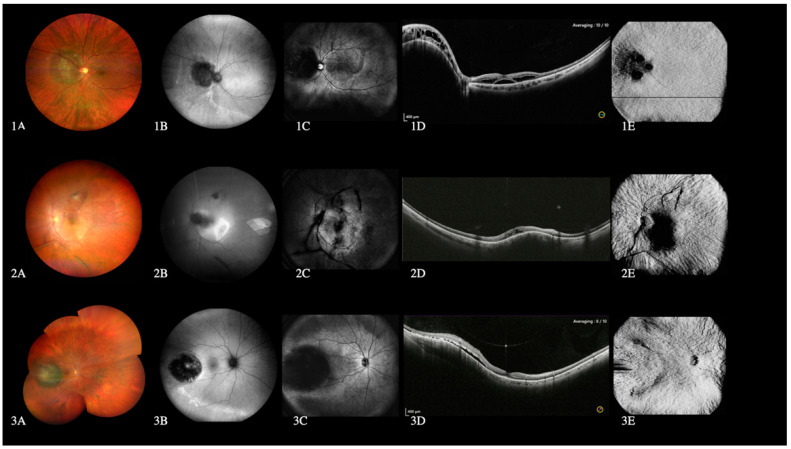
Images from left to right: color fundus retinography, green-autofluorescence, scanner laser ophthalmoscopy (SLO) image, wide-field optical coherence tomography (WF-OCT), and wide-field optical coherence tomography angiography (WF-OCTA). Choroidal hemangioma adjacent to the optic nerve is shown at the top (**1A**–**1E**), choroidal granuloma secondary to tuberculosis in the middle (**2A**–**2E**), and choroidal melanoma at the bottom (**3A**–**3E**). A dark image on the SLO is observed in hemangioma (**1C**) and melanoma (**3C**). WF-OCT shows intraretinal and subretinal fluid in the hemangioma (**1D**), intraretinal fluid in the granuloma (**2D**) and subretinal fluid in the melanoma (**3D**). Vascularization can be seen on the WF-OCTA in the hemangioma (**1E**) and melanoma (**3E**) but not in the granuloma (**2E**).

WF-OCT diagnosed the choroidal lesions providing information on their size, shape, distance from the optic nerve and macula, and impact on the choriocapillaris and choroid. Indeed, it revealed no vascularity in benign nevi and tubercular granuloma but dense vascularity in melanoma and choroidal hemangioma. The SLO image appeared dark in melanoma and grey in hemangioma and benign nevi. Furthermore, the granulomas’ SLO image was lighter than the surrounding tissue in benign nevi and comparable to the shade of the surrounding tissue in granulomas (Figure 4).

WF-retinography diagnosed the choroidal lesions providing additional information on size, shape, distance from optic nerve and macula, color, and the presence of concomitant findings such as drusen of pigment alterations.

### 3.6. Optic Nerve Lesions

**Figure 5:**
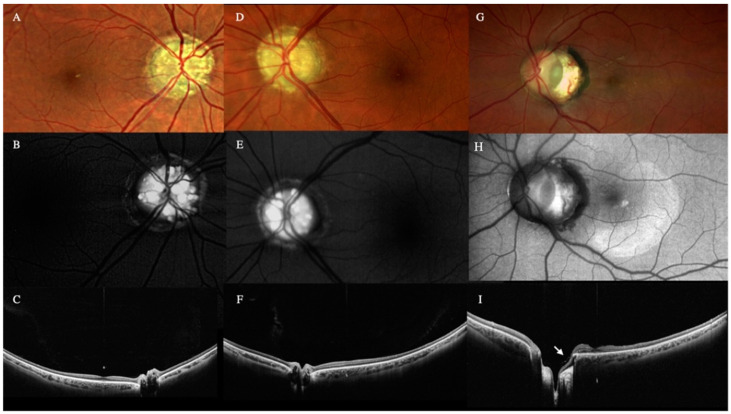
Optic nerve color photo (**A**,**D**) and autofluorescence (**B**,**E**) of a patient with bilateral optic nerve drusen (OND). Notice the hyper-autofluorescence in both optic nerves. Wide-field (WF) and deep field (DF) optical coherence tomography (OCT) radial scan centered on the optic nerve (**C**,**F**) of the right and left eye. OCT shows the OND as oval hyporeflective lesions with hyperreflective borders. Optic nerve retinography (**G**) and autofluorescence (**H**) of a temporal optic nerve coloboma. WF-OCT image (**I**) shows the herniation of the retina into the colobomatous area. This patient underwent a vitrectomy and internal limiting membrane (ILM) flap to treat the retinal detachment. Notice the ILM flap (arrow) and the absence of retinal detachment five years after the surgery.

Optic nerve drusen (OND) were detected with WF OCT as low-reflective multiple ovoid lesions with hyperreflective borders in the optic nerve head (Figure 5). Despite being also seen with color retinography and with standard OCT, only WF OCT could show the depth of the lesions into the optic nerve.

The WF OCT showed the optic pit as a tubular excavation into the optic nerve, visualizing the secondary retinal detachment in a single scan and the vitreous strands connected to the optic disc pit.

Although only WF OCT could show the depth of the lesions into the optic nerve, optic pit and coloboma could also be detected with color retinography and standard OCT. Specifically, retinal tissue herniated into the colobomatous area was visualized in optic nerve coloboma cases (Figure 5).

### 3.7. Difficult Situations

#### 3.7.1. Miosis

Eyes whose fundus could not be inspected with slit lamp biomicroscopy or standard OCT due to the narrowed angle configuration, allergy to mydriatics, posterior synechiae, or dilation refusal were analyzed with WF-OCT and WF retinography.

#### 3.7.2. Media Opacities

WF-OCT images were obtained in 10 eyes with corneal opacity and 20 eyes with lens cloudiness (cataract, intraocular lens opacification, or posterior capsule opacification). WF-retinography could partially inspect the fundus of those eyes but not slit lamp biomicroscopy or standard OCT.

#### 3.7.3. Pediatric Cases

**Figure 6:**
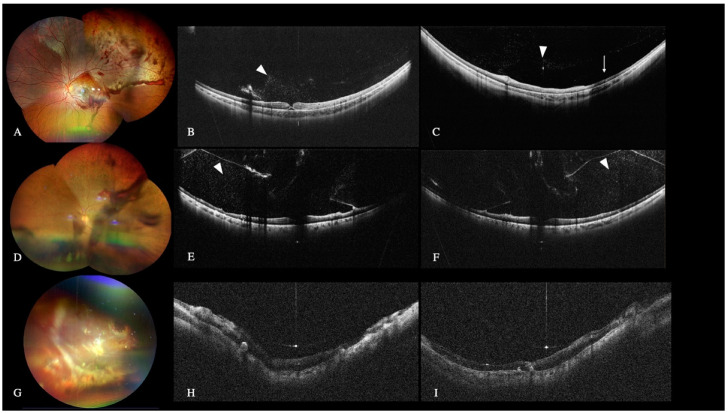
WF color fundus retinography (**A**) of an 11-year-old boy showed vitreous and preretinal hemorrhage (VH) and traumatic macular hole three days after contusive trauma with a tennis ball. Despite the VH, high-quality wide-field (WF) optical coherence tomography (OCT) images could be obtained (**B**,**C**). The VH was observed as mild, diffuse white dots in the vitreous chamber (arrowheads), and the formation of a macular hole can be observed. Only the external retinal layer is still continuous (**C**). The WF-OCT shows an area of peripheral retinal thinning (arrow) and confirmed neither detachment nor other retinal breaks, even in the most peripheral area. WF color fundus retinography of a vitreous hemorrhage in a case of proliferative diabetic retinopathy (**D**). WF-OCT shows VH, although some posterior shadows onto the retina can be noticed. The vitreous cortex appears partially detached, taut, and thickened (arrowheads) (**E**,**F**). Color fundus retinography (**G**) of an 8-year-old boy with Peter’s anomaly, who underwent a second perforating keratoplasty and vitreous surgery for retinal detachment relapsed with proliferative vitreoretinopathy (PVR) in his left eye. The child presented corneal opacity, and despite the partial and challenging visualization of the fundus on the retinography, WF-OCT could be obtained to confirm that the retina was wholly attached (**H**,**I**).

**Figure 7:**
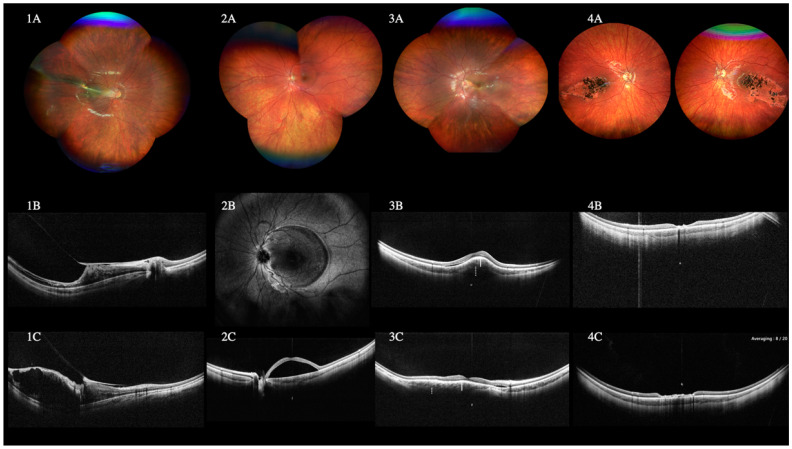
A case of familial exudative vitreoretinopathy (FEVR) in a 4-year-old boy is shown in the wide-field (WF) fundus color image (**1A**), showing temporal vessels rectification, macula and papilla displacement, and a macular fold. Wide-field optical coherence tomography (OCT) shows the retinal tractions and folds (**1B**,**1C**). WF fundus color image (**2A**), scanner laser ophthalmoscopy (SLO) image (**2B**), and WF-OCT (**2C**) of a 10-year-old girl with a large macular detachment secondary to an optic pit. The perimeter of the retinal detachment is delimitated on the SLO image (**2B**). In one scan, we can appreciate the entire macular detachment, the depth of the pit, and the vitreous strand in the pit (**2C**)—a 9-year-old girl affected by tuberous sclerosis presented with a choroidal lesion. The WF color fundus image (**3A**) shows irregular elevations at the posterior pole. The WF-OCT (**3B**,**3C**) showed a minimal extrafoveal serous detachment of the superior macula, a thickened choroid (white line), and new lesions with irregular shape under the choroid (white dotted line). A diagnosis of subchoroidal granuloma compatible with neurofibromatosis was made. A 4-year-old boy was diagnosed with retinopathy of prematurity (ROP), showing a bilateral macular irregular, an atrophic area with multiple pigmented dots in the fundus color image (**4A**, right and left eye). The WF-OCT of the right (**4B**) and the left eye (**4C**) confirmed the atrophy of all the macular layers with the normal choroid.

Thirty-seven eyes of patients of pediatric age (≤18 years) were analyzed. The mean age was 11.4 (range 5–17). The fundus could be inspected with WF-retinography. However, the slit lamp biomicroscopy and standard OCT could partially examine the same findings. Figure 6 and Figure 7 report two challenging pediatric cases of an 11-year-old boy who showed vitreous and preretinal hemorrhage (VH) and traumatic macular hole three days after contusive trauma with a tennis ball and a case of familial exudative vitreoretinopathy (FEVR) in a 4-year-old boy.

#### 3.7.4. Postoperative Follow-Up

During the first week after vitreoretinal surgery, ninety eyes were examined as part of a follow-up examination. A tamponade (air, gas, or silicone oil) was used to replace the removed vitreous. The OCT-S1 image was obtained, allowing the assessment of the fundus that had not been covered by air/gas.

Residual subretinal fluid and the location of small breaks could be detected in cases of persistent or recurrent detachment. WF-retinography could inspect the fundus of the same eyes, but slit lamp biomicroscopy and standard OCT could only inspect it partially.

### 3.8. Role of WF and UWF OCTA

**Figure 8:**
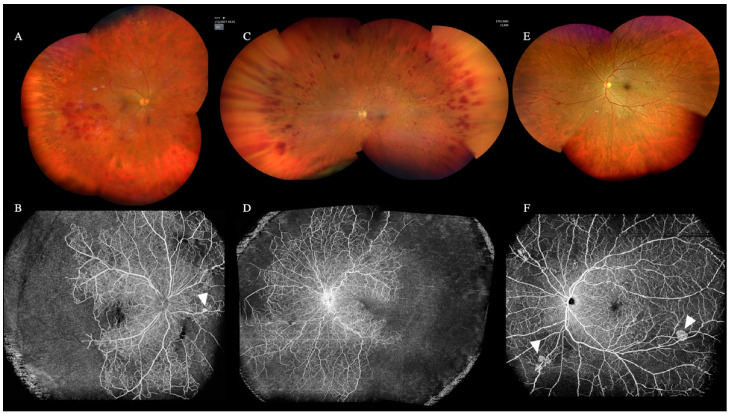
Ultra-wide-field (UWF) color fundus retinography (**A**,**C**,**E**), wide-field optical coherence tomography angiography (WF-OCTA) (**B**,**F**), and UWF-OCTA created with the mosaic software (**D**). (**A**,**C**) show two cases of central retinal vein occlusion (CRVO). The WF-OCTA images (**B**,**D**) revealed peripheral retinal ischemia with a nasal neovascularization bud in (**B**) (arrowheads) and without neovascularization in (**D**). (**E**) shows an eye with diabetic retinopathy and the corresponding WF-OCTA (**F**) manifested mild peripheral ischemia and neovascularization elsewhere (NVE) (arrowheads).

WF-OCTA was performed in 595 eyes (40%), and UWF-OCTA was deployed to analyze a subgroup of only 41 eyes (3%) (Figure 8).

WF-OCTA could detect ischemic areas and new vessels in eyes with vein or artery occlusions. When the B-scan was combined with WF-OCTA, neovascularization (NV) as preretinal vessels and intraretinal shunts as intraretinal vessels were easily distinguished.

## 4. Discussion

In this study, we report the advantages of WF- and UWF-OCT and WF- and UWF OCTA in managing different vitreoretinal diseases using the new WF SS-OCT Xephilio S1 as well as the “effectiveness” of WF- and UWF-OCT and OCTA in diagnosing vitreoretinal diseases whose diagnosis could not be achieved with any other clinical or instrumental examination in a real-life setting. The results were analyzed per type of lesions and conditions encountered.

### 4.1. Vitreous Pathologies

Despite being easily detected with slit lamp biomicroscopy, vitreous floaters evaluation through color retinography is still challenging.

In our study, the combination of color SLO, WF, and deep field (DF) OCT proved to be a useful diagnostic tool for vitreous floaters, as reported by previous studies [16,17].

Indeed, floaters were clearly detected as hyper-reflective spots with non-homogenous patterns on OCT and dark shadows on the WF-SLO image, regardless of the symptoms. WF-OCT detected the physiologic PVD and its features, such as the detachment extension and the location of residual vitreoretinal adhesions. Despite being easily diagnosed with ocular ultrasounds (US), the WF-OCT may quickly help detect the PVD in less time than the US. In addition, the ocular US requires more cooperative patients and skilled technicians as it may be challenging to differentiate a retinal from a vitreous detachment, depending on the angle of visualization [18]. However, further comparative studies are required. Notably, PVD was present in only 66% of patients with symptomatic floaters. Therefore, PVD diagnosis can be routinely overrated with standard fundus examination. 

WF-OCT detected several vitreous opacities. Despite fairly different OCT patterns (AH cellularity was bigger than inflammatory and red blood cells), no definitive diagnosis could have been provided to differentiate the findings across the images. Indeed, the vitreous opacities appeared as hyperreflective aggregated dots in all images. Therefore, the fundus examination or retinography is still necessary to distinguish the nature of cellularity.

### 4.2. Peripheral Retinal Lesions and Retinal Detachment

Only a subgroup of 311 eyes was analyzed with UWF-OCT due to patients’ cooperation and the length of the examination. Indeed, while a central radial 23 mm takes less than 10 s, a UWF-OCT with a complete raster scan of 8 peripheral areas takes 10 min. In this subgroup, the occurrence of significant peripheral lesions not visible with slit lamp biomicroscopy (with and without contact lenses) nor with WF or UWF retinography was detected in 39% of eyes. Indeed, in a case series of 125 eyes, Sodhi et al. found that eighty-six out of 125 eyes (69%) had peripheral retinal lesions. Therefore, the UWF-OCT can provide further information on the area that cannot be visualized by standard OCT devices with a 50-degree field of view, thus providing a holistic clinical picture, which can potentially aid in understanding the various retinal pathologies [19].

Furthermore, one case of shallow retinal detachment was detected. Regarding the rhegmatogenous retinal detachment, WF and UWF-OCT showed the detachment’s location and extension, the retinal breaks’ location, and the presence of preretinal membranes. In addition, peripheral retinal imaging helped in discriminating limited areas of retinoschisis in a high number of eyes, in the differential diagnosis between retinal detachment and retinoschisis, and further assessing the location of inner or outer breaks in the retinoschisis. All features observed are consistent with previous reports, including relative thinning of all nuclear layers and the attenuation of choroidal vessels [14].

### 4.3. High Myopia

Although high-resolution three-dimensional magnetic resonance imaging (3D-MRI) could be an excellent research tool for deepening our understanding of myopia, it is not practical for routine clinical care due to its high cost and limited availability. The recently introduced WF and UW technology greatly visualize high myopic eyes (axial length ≥ 26.5 mm), whose irregular eyewall and staphyloma profile are often difficult to visualize in one exam alone. Indeed, the new OCT device allowed the 3D-maps reconstruction of posterior staphylomas by using multiple scan lines. A recent study by Shinohara et al. involving 100 highly myopic eyes with a mean axial length of 30.2 ± 2.1 mm showed that the UWF SS-OCT provided results according to those found with 3D MRI in detecting posterior staphylomas. In addition, they detected a spatial relation between myopic retinoschisis and staphylomas [14,20].

According to the previous reports, the wide-field technology helps provide peripheral retinal lesions details even in “only-macular” diseases such as MTM, thus aiding a holistic picture of the myopic eye. However, despite the great results, further studies are required to deeply analyze the role of peripheral retinal visualization in managing MTM [21].

### 4.4. Choroidal Lesions

The DF images offered an excellent visualization of the choroid. WF-OCT allowed assessment of the size, shape, and extension of choroidal lesions and macula and the intraretinal or subretinal fluid effect on choroid and choriocapillaris [13].

In our study, the WF-OCTA showed the presence or absence of tumor vascularization, although we could not find specific patterns of vascularization nor differentiate one choroidal lesion from others. 

Although the US and clinical ophthalmoscopy still represents the gold standard in detecting ocular tumors [22], wide-field OCTA is increasingly playing an important role in visualizing choroidal vasculature, especially in situations in which serial imaging could be beneficial in follow-up and management of the disease. Moreover, offering WF color fundus retinography, WF autofluorescence in one consultation alone, WF-OCT, and OCTA may represent an adjunctive tool in ocular tumor management [23].

### 4.5. Optic Nerve Lesions

Alongside macular structure, the DF images of the optic nerve allowed us to visualize optic nerve anomalies and secondary retinal detachments. The role of WF and UWF imaging in managing optic nerve lesions is controversial. Despite the SS-OCT providing plenty of information, wide-field retinal imaging could help detect retinal nerve fiber layer (RNFL) changes in patients with glaucoma. Indeed, Lee et al. first evaluated the diagnostic ability of wide-field NRFL maps with SS-OCT to detect preperimetric and early perimetric glaucoma. In their study involving one hundred eighty-four eyes, the wide-field RNFL thickness map showed a high sensitivity in preperimetric glaucoma and early-perimetric diagnosis [24]. Nonetheless, further studies are required to investigate the role of WF and WF-OCT in optic nerve diseases.

### 4.6. Difficult Situations

The imaging acquisition of pediatric, high myopic, non-cooperative patients or patients’ eyes who recently underwent vitreoretinal surgery is often challenging. Indeed, the long acquisition time and the pupillary dynamic status may prevent a reliable diagnosis. The new WF- and UWF technology, whose scan time is less than a minute and requires a minimum of 2.5 mm pupil size, may overcome these limitations. 

In our case series, the WF and UWF had a significant role in pediatric cases. To be specific, this technology played a crucial role in an 11-year-old boy case with a 3-day history of contusive trauma, which had caused vitreous and preretinal hemorrhage and an atraumatic macular hole. Despite the VH preventing a reliable ophthalmoscopy diagnosis, the high-quality WF-OCT images clearly showed a macular hole alongside the VH. 

Furthermore, the WF- and UWF technology helped assess an 8-year-old boy with Peter’s anomaly who had previously undergone perforating keratoplasty and vitreous surgery for retinal detachment. Despite the severe corneal opacity, the WF-OCT could be obtained, confirming that the retina was completely attached. 

According to Kothari et al., widespread WF- and UWF- technology may be crucial, especially in pediatric diseases that cause irreversible blindness, such as retinopathy of prematurity and Coat’s disease. Indeed, it overcomes several limitations of other imaging methods, such as sedation and intravenous dye injection [25]. Moreover, as previously demonstrated by Athikarisamy et al., performing quality wide-field imaging may also help in an off-site telehealth diagnosis of all patients with referral-warranting pediatric retinal diseases [26].

Moreover, due to a faster scan and great depth of focus, the WF and UWF technology may allow the detection of retinal images in eyes with nystagmus or in patients with intellectual disabilities.

Regarding the early postoperative follow-up, ninety eyes were analyzed during the first week after vitreoretinal surgery. Due to the considerable depth of focus, the UWF system allowed the peripheral retina, intraocular gas bubble, and posterior pole to be simultaneously in focus in gas-filled eyes, allowing us to inspect the fundus not covered by air/gas and assess the residual subretinal fluid and the location of small breaks in recurrent retinal detachments [26].

### 4.7. WF and UWF OCTA

Despite acquiring less than 40 patients, WF and UWF OCTA helped provide information regarding the peripheral vascular pattern in specific diseases.

As previously described for choroidal lesions, this technology may implement the standard clinical practice for widespread diseases such as diabetic retinopathy and vein occlusions [27,28]. Hirano et al. found a higher sensitivity and specificity than fluorescein angiography (FA) in detecting non-perfusion area (NPA) and neovascularization (NV) [27]. Accordingly, by combining the B-scan with the WF-OCTA in our series, we detected NPA and distinguished the NV from intraretinal shunts.

Furthermore, a significant correlation between FA and WF-OCTA was found by Shiraki in analyzing the branch retinal vein occlusions’ NPA. Indeed, the images showed a significant association with vascular length by using a separate regression analysis of the areas of retinal nonperfusion in FA and the total FA. In addition, the WF-OCTA may help detect NV that could not be clinically evaluated, as previously demonstrated by Huemer et al., that reported an additional 31% detection of NV [29].

## 5. Conclusions

In summary, the information acquired with WF, UWF OCT, and OCTA helped manage 298 eyes (20%), representing the only technology that allowed a reliable examination of challenging cases such as miosis, severe media opacity, pediatric patients, and postoperative follow-up. In our case series, the WF and DP images helped assess several lesions that could not be investigated by enrolling in any different technology, further proving underrated features of the retina and choroid. Moreover, without involving long lasting and painful image acquisition protocols, this imaging technology enables faster management and increases patient compliance, providing reliable diagnoses that do not require further investigations.

Despite the advantages, several limitations have been found across the analysis. First, the processing time for acquiring and analyzing raster scans is still longer. Second, the mean acquisition time for cooperative patients with good visual function can be lengthened depending on the visual disturbances and the fixation capacity, which allowed us to perform UWF-OCT and OCTA in a selected number of patients. Third, the motion artifacts, more common at the periphery of the retina and in non-compliant patients, can lead to misalignment and undocumented areas in the B-scans, 3D reconstructions, and OCTA mosaics. Fourth, due to a high time in acquisition and mosaic creation in OCTA, we advise performing this exam only in eyes with vascular diseases. Fourth, the OCTA imaging detection could be problematic in high myopic eyes because of an unreliable segmentation. Fifth, the costs and the limited availability of the WF and UWF technologies restrict their use in clinical practice. Sixth, while all patients underwent widefield OCT, much smaller percentages underwent imaging with the other techniques. Finally, we did not compare our findings with those obtained with other retinal imaging modalities such as the Optos UWF apparatus (Optos Daytona, Optos PLC, Dunfermline, UK). Despite these limitations, WF and UWF OCT and OCTA imaging helped manage a significant number of cases, becoming an indispensable tool for the high-quality management of patients.

## Figures and Tables

**Table 1:** Patients’ demographics.

Features	Number (%)
Sex:	768
*Male*	358 (46.61%)
*Female*	410 (53.39%)
Age (years); (mean-range)	59.6 (5–94)
Eye involved	1472
*Right*	735 (49.93%)
*Left*	737 (50.07%)

**Table 2:** Diagnosis features.

Diagnosis	Number, N = 1472 Eyes
**High myopia**	**329**
Myopic Tractional Maculopathy (MTM)	91
Macular buckle	37
**Retinal detachment**	**144**
Follow up	108
Primary detachment	36
**Peripheral lesions**	**122**
Retinal tears	12
Peripheral retinoschisis	63
Other peripheral lesions	47
**Vascular retinal diseases**	**60**
BRVO/CRVO	26
CRAO	2
Diabetic retinopathy	32
**Pediatric cases**	**37**
Normal	16
Best dystrophy	4
RRD associated with Peter’s anomaly	2
FEVR	2
ROP	2
Diffuse choroidal hemangioma in Sturge Weber syndrome	2
Combined hamartoma of the retina and RPE	2
Incontinentia pigmenti	2
Morning glory syndrome	1
Traumatic FTMH with VH and traumatic retinopathy	1
Traumatic retinal detachment	1
Retinal detachment associated with Marfan syndrome	1
Optic disc pit maculopathy	1
**Choroidal lesions**	**16**
Choroidal granuloma	1
Choroidal melanoma	4
Choroidal nevus	4
**Circumscribed choroidal hemangioma**	**3**
Diffuse choroidal hemangioma	2
Combined hamartoma of the retina and RPE	2
**Vitreomacular Traction (VMT) syndrome**	**10**
**Optic nerve pathologies**	**8**
Optic nerve coloboma	1
Optic nerve drusen	4
Optic disc pit	3
**Uveitis**	**5**
Toxoplasma retinitis	3
Behçet disease	2

Abbreviations: Myopic Tractional Maculopathy: MTM; Branch Retinal Vein Occlusion: BRVO; Central Retinal Vein Occlusion: CRVO; Central Retinal Artery Occlusion: CRAO; Rhegmatogenous Retinal Detachment: RRD; Familial Exudative Vitreoretinopathy: FEVR; Retinopathy of Prematurity: ROP; Retinal Pigment Epithelium: RPE; Full Thickness Macular Hole: FTMH; Vitreous Hemorrhage: VH; Vitreomacular Traction: VMT; Number: n.

**Table 3:** Time of wide-field imaging acquisition in cooperative patients and those with good visual acuity.

Modality of Scan	Time of Acquisition (Seconds)
Radial 23 mm	7 s
Radial HD 23 mm	14 s
3 D Cube 23 mm	14 s
Choroid	14 s
OCTA 23 × 20 mm	30 s
NDB:	
Macular 3D	3 s
Glaucoma 3D	2 s
Disc 3D	3 s

Abbreviations: High definition: HD, Optical coherence tomography angiography: OCTA, Normative database: NDB, Millimeters: mm, Seconds: s, Three-dimensional: 3D.

## References

[1] Aumann S., Donner S., Fischer J., Müller F. (2019). Optical Coherence Tomography (OCT): Principle and Technical Realization. High Resolution Imaging in Microscopy and Ophthalmology.

[2] Spaide R.F., Fujimoto J.G., Waheed N.K., Sadda S.R., Staurenghi G. (2018). Optical Coherence Tomography Angiography. Prog. Retin. Eye Res..

[3] Huang D., Swanson E.A., Lin C.P., Schuman J.S., Stinson W.G., Chang W., Hee M.R., Flotte T., Gregory K., Puliafito C.A. (1991). Optical Coherence Tomography. Science.

[4] Choma M., Sarunic M., Yang C., Izatt J. (2003). Sensitivity Advantage of Swept Source and Fourier Domain Optical Coherence Tomography. Opt. Express.

[5] de Boer J.F., Cense B., Park B.H., Pierce M.C., Tearney G.J., Bouma B.E. (2003). Improved Signal-to-Noise Ratio in Spectral-Domain Compared with Time-Domain Optical Coherence Tomography. Opt. Lett..

[6] Leitgeb R., Hitzenberger C., Fercher A. (2003). Performance of Fourier Domain vs. Time Domain Optical Coherence Tomography. Opt. Express.

[7] Klein T., Wieser W., Reznicek L., Neubauer A., Kampik A., Huber R. (2013). Multi-MHz Retinal OCT. Biomed. Opt. Express.

[8] Fujimoto J., Swanson E. (2016). The Development, Commercialization, and Impact of Optical Coherence Tomography. Investig. Ophthalmol Vis. Sci..

[9] Wang Y., Nelson J., Chen Z., Reiser B., Chuck R., Windeler R. (2003). Optimal Wavelength for Ultrahigh-Resolution Optical Coherence Tomography. Opt. Express.

[10] Grulkowski I., Liu J.J., Potsaid B., Jayaraman V., Lu C.D., Jiang J., Cable A.E., Duker J.S., Fujimoto J.G. (2012). Retinal, Anterior Segment and Full Eye Imaging Using Ultrahigh Speed Swept Source OCT with Vertical-Cavity Surface Emitting Lasers. Biomed. Opt. Express.

[11] Potsaid B., Gorczynska I., Srinivasan V.J., Chen Y., Jiang J., Cable A., Fujimoto J.G. (2008). Ultrahigh Speed Spectral/Fourier Domain OCT Ophthalmic Imaging at 70,000 to 312,500 Axial Scans per Second. Opt. Express.

[12] Baldascino A., Ripa M., Carlà M.M., Caporossi T., Grieco G., Gambini G., de Vico U., Raguso G., Kilian R., Rizzo C. (2022). Optical Coherence Tomography Angiography to Estimate Early Retinal Blood Flow Changes after Uncomplicated Cataract Surgery. Vision.

[13] Nagiel A., Lalane R.A., Sadda S.R., Schwartz S.D. (2016). Ultra-Widefield Fundus Imaging: A Review of Clinical Applications and Future Trends. Retina.

[14] Choudhry N., Golding J., Manry M.W., Rao R.C. (2016). Ultra-Widefield Steering-Based Spectral-Domain Optical Coherence Tomography Imaging of the Retinal Periphery. Ophthalmology.

[15] Parolini B., Palmieri M., Finzi A., Besozzi G., Lucente A., Nava U., Pinackatt S., Adelman R., Frisina R. (2021). The New Myopic Traction Maculopathy Staging System. Eur. J. Ophthalmol..

[16] Son G., Sohn J., Kong M. (2021). Acute Symptomatic Vitreous Floaters Assessed with Ultra-Wide Field Scanning Laser Ophthalmoscopy and Spectral Domain Optical Coherence Tomography. Sci. Rep..

[17] Milston R., Madigan M.C., Sebag J. (2016). Vitreous Floaters: Etiology, Diagnostics, and Management. Surv. Ophthalmol..

[18] Lahham S., Ali Q., Palileo B.M., Lee C., Fox J.C. (2019). Role of Point of Care Ultrasound in the Diagnosis of Retinal Detachment in the Emergency Department. Open Access Emerg. Med. OAEM.

[19] Sodhi S.K., Golding J., Trimboli C., Choudhry N. (2021). Feasibility of Peripheral OCT Imaging Using a Novel Integrated SLO Ultra-Widefield Imaging Swept-Source OCT Device. Int. Ophthalmol..

[20] Shinohara K., Tanaka N., Jonas J.B., Shimada N., Moriyama M., Yoshida T., Ohno-Matsui K. (2018). Ultrawide-Field OCT to Investigate Relationships between Myopic Macular Retinoschisis and Posterior Staphyloma. Ophthalmology.

[21] Li S., Li T., Wang X., Cai X., Lu B., Chen Y., Liu C., Wu Q. (2021). Natural Course of Myopic Traction Maculopathy and Factors Influencing Progression and Visual Acuity. BMC Ophthalmol..

[22] Wolff-Korman P.G., Kormann B.A., Hasenfratz G.C., Spengel F.A. (1992). Duplex and Color Doppler Ultrasound in the Differential Diagnosis of Choroidal Tumors. Acta Ophthalmol..

[23] Callaway N.F., Mruthyunjaya P. (2019). Widefield Imaging of Retinal and Choroidal Tumors. Int. J. Retin. Vitr..

[24] Lee W.J., Na K.I., Kim Y.K., Jeoung J.W., Park K.H. (2017). Diagnostic Ability of Wide-Field Retinal Nerve Fiber Layer Maps Using Swept-Source Optical Coherence Tomography for Detection of Preperimetric and Early Perimetric Glaucoma. J. Glaucoma.

[25] Kothari N., Pineles S., Sarraf D., Velez F., Heilweil G., Holland G., McCannel C.A., Onclinx T., McCannel T.A., Sadda S.R. (2019). Clinic-Based Ultra-Wide Field Retinal Imaging in a Pediatric Population. Int. J. Retin. Vitr..

[26] Athikarisamy S.E., Lam G.C., Ross S., Rao S.C., Chiffings D., Simmer K., Bulsara M.K., Patole S. (2020). Comparison of Wide Field Imaging by Nurses with Indirect Ophthalmoscopy by Ophthalmologists for Retinopathy of Prematurity: A Diagnostic Accuracy Study. BMJ Open.

[27] Hirano T., Kakihara S., Toriyama Y., Nittala M.G., Murata T., Sadda S. (2018). Wide-Field En Face Swept-Source Optical Coherence Tomography Angiography Using Extended Field Imaging in Diabetic Retinopathy. Br. J. Ophthalmol..

[28] Pichi F., Smith S.D., Abboud E.B., Neri P., Woodstock E., Hay S., Levine E., Baumal C.R. (2020). Wide-Field Optical Coherence Tomography Angiography for the Detection of Proliferative Diabetic Retinopathy. Graefes Arch. Clin. Exp. Ophthalmol..

[29] Huemer J., Khalid H., Wagner S.K., Nicholson L., Fu D.J., Sim D.A., Patel P.J., Balaskas K., Rajendram R., Keane P.A. (2021). Phenotyping of Retinal Neovascularization in Ischemic Retinal Vein Occlusion Using Wide Field OCT Angiography. Eye.

